# Incidence and Microbiology of Hospital-Acquired Infections in COVID-19 Patients between the First and the Second Outbreak of the SARS-CoV-2 Pandemic: A Retrospective, Observational Study

**DOI:** 10.3390/microorganisms10122372

**Published:** 2022-11-30

**Authors:** Corti Nicolò, Tordato Federica, Guendalina De Nadai, Mapelli Sarah, Garlanda Cecilia, Pocaterra Daria, Casana Maddalena, Bonfanti Paolo, Morelli Paola

**Affiliations:** 1 Infectious Disease Unit, San Gerardo Hospital, 20900 Monza, Italy; 2 Residency School in Infectious and Tropical Diseases, University of Milano-Bicocca, 20900 Monza, Italy; 3 Department of Biomedical Sciences, Humanitas University, Pieve Emanuele, 20072 Milan, Italy; 4 Infectious Disease Unit, IRCCS Humanitas Research Hospital, Rozzano, 20089 Milan, Italy; 5 Emergency Department, IRCCS Humanitas Research Hospital, Italy IRCCS Humanitas Research Hospital, Rozzano, 20089 Milan, Italy; 6 Residency School in Emergency Medicine, Humanitas University, Pieve Emanuele, 20072 Milan, Italy; 7 IRCCS Humanitas Research Hospital, Rozzano, 20089 Milan, Italy; 8 Department of Medicine and Surgery, University of Milano-Bicocca, 20090 Monza, Italy

**Keywords:** hospital-acquired infections (HAIs), multi-drug resistance, COVID-19, antimicrobial stewardship

## Abstract

With almost 638 million cases and over 6 million deaths worldwide, the SARS-CoV-2 pandemic represents an unprecedented healthcare challenge. Although the management and natural history of COVID-19 patients have changed after the introduction of active therapies and vaccination, the development of secondary infections complicates hospital stay. This is a single-center, retrospective, observational study that explores the incidence and microbiology of hospital-acquired infections (HAIs) in two subsequent populations of hospitalized patients with COVID-19. Demographic, pre-hospitalization baseline characteristics, therapeutic options and microbiology data about secondary infections were collected for a total of 1153 cases. The second population appeared to have a higher median age (73 vs. 63 years, respectively), comorbidities (median Charlson Comorbidity Index Score was 4 vs. 1, respectively) and incidence of secondary infections (23.5% vs. 8.2%) with respect to the first. A higher incidence of multi-drug resistant organisms (MDROs), including difficult-to-treat resistant (DTR) *Pseudomonas*, methicillin-resistant *Staphylococcus aureus* (MRSA) and vancomycin-resistant *Enterococcus* (VRE), was also observed. Both patients’ characteristics and poor adherence to standard hygiene and infection control protocols may have contributed to the higher incidence of these events and may have impacted on the natural history of the disease. In-hospital mortality rates were similar, despite the introduction of active therapies against COVID-19 (24.7% vs. 23.5%, respectively). The incidence of HAIs may have contributed to the unchanged mortality and prompts for more effective antimicrobial stewardship and infection control procedures in COVID-19.

## 1. Introduction

After initial cases in Wuhan, China, in late December 2019, the severe acute respiratory syndrome coronavirus 2 (SARS-CoV-2), the causative agent of coronavirus-disease 2019 (COVID-19), spread rapidly all over the world [1]. Virus transmission occurs primarily via respiratory droplets from infected individuals: a minority of cases are associated with airborne transmission and indirect contact with contaminated surfaces [2]. The natural history of the disease varies from asymptomatic infection to severe and life-threatening pneumonia conditioning respiratory failure [3]. COVID-19 is also associated to a high risk of thromboembolic events [4,5]. Several risk factors have been associated to increased odds of morbidity and mortality; of note, advanced age and the presence of chronic and degenerative diseases play an important part [3,6,7]. Hospitalized COVID-19 patients appear to be at increased risk of developing secondary infections [8,9,10]. This study provides a comparison of hospitalized COVID-19 patients between the first and the second outbreak of the pandemic, focusing on a descriptive characterization of related secondary infections. As a matter of fact, viral illnesses per se, such as influenza, are linked to a high risk of developing a secondary bacterial infection, especially under severe or critical clinical conditions [11]. The incidence of secondary infections may be particularly true for hospitalized patients in an emergency setting, where shortage and lack of adequate infection control and prevention procedures may favor transmission of bacteria within hospitals, either directly among hospitalized patients or indirectly among healthcare workers and healthcare equipment, such as intravascular devices, catheters, and ventilators [12,13]. Outbreaks of MDROs in COVID-19 patients have been reported in several studies and different factors have been associated to the incidence of these events [14,15,16,17]: comorbidities, immune suppression associated with SARS-CoV-2 infection and with the critical illness per se, the use of steroids, and the frequent need for invasive life support procedures predispose patients with COVID-19 to a high risk of MDRO-associated HAIs [18,19,20]. In most scenarios, secondary lung infections (hospital-associated pneumonia and ventilator-associated pneumonia, HAP and VAP, respectively), urinary tract infections (UTIs), and bloodstream infections (BSIs) have been reported [9,10,19]. The most frequently isolated microorganisms in COVID-19-related HAIs include typical nosocomial pathogens with a high antibiotic-resistance profile, such as methicillin-resistant *Staphylococcus aureus* (MRSA), vancomycin-resistant *Enterococcus* spp. (VRE), carbapenem-resistant *Enterobacteriaceae* (CRE), and *Pseudomonas aeruginosa* [9,10,13,17,21]. The prevalence of the different multi-drug resistant pathogens changes according to local epidemiology and hospital settings [22]: several determinants have been recognized as risk factors for MDRO infection, such as a history of antibiotic use, residence in a long-term care facility, a history of hospitalization, male sex, and older age (> 65 years) [23,24,25]. Because the exact role of secondary infections in shaping the natural history of COVID-19 has not been defined yet, understanding whether and how COVID-19 patients are at risk of developing secondary infections is crucial. Moreover, defining the epidemiology of these events may provide better patient care and reinforce antimicrobial stewardship programs to efficaciously manage antibiotic-resistant bacteria, allowing for rationale antibiotic use and effective infection control. In line with these aspects, considering how the management of COVID-19 patients has changed over time due to a better understanding of the disease [26,27], this study aims at comparing two populations of hospitalized COVID-19 patients between the first and the second outbreak of the pandemic in a university hospital in Milan, Italy. By providing a qualitative and quantitative description of secondary infections, this study aims at explaining possible factors associated to their incidence.

## 2. Materials and Methods

### 2.1. Design and Ethics

This is a single-center, retrospective, observational study conducted at the IRCCS Humanitas Clinical and Research Hospital in Milan, Italy. In light of the retrospective nature of the study, with respect to patients’ privacy and the sole use of data that were collected for routine and clinical practice, an ethical committee approval and a patient’s informed consent were not required. All data were collected and described anonymously. Informed consent for general hospital care is routinely signed by all patients that are admitted to the hospital.

### 2.2. Study Cohort

All consecutive patients who were hospitalized at the Humanitas Clinical and Research Hospital during 1 March 2020–30 April 2020 (first population) and 1 October 2020–15 December 2020 (second population), with clinical and epidemiological confirmation for COVID-19, were enrolled in the study. A total of 1153 patients were analyzed, comprising 510 patients from the first population and 643 from the second population. In particular:

- All cases were confirmed via viral nucleic acid detection using RT-PCR of upper (nasopharyngeal) and/or lower (bronchoalveolar lavage [BAL]) respiratory tract specimens.
- Testing was carried out in the microbiology laboratory at Humanitas Clinical and Research Hospital.

Probable cases, presenting epidemiological, clinical, and/or radiological features compatible with SARS-CoV-2 infection, for which laboratory testing was inconclusive or negative, were excluded from the analysis.

### 2.3. Screening for MDROs

Active monitoring of MDRO-associated infection is performed for all patients admitted to the intensive care unit at Humanitas Clinical and Research Hospital. Rectal and nasal swabs are sampled out at ICU admission for carbapenem-resistant microorganisms (CRE, *A. baumannii*, *Pseudomonas* spp.) and MRSA, respectively. Antibiotic susceptibility and mechanisms of resistance are confirmed by the microbiology lab at Humanitas Clinical and Research Hospital and patterns of resistance are interpreted according to EUCAST breakpoints for antimicrobial susceptibility [28].

### 2.4. Data Collection

Electronic medical records of patients with a laboratory-confirmed SARS-CoV-2 infection were retrospectively reviewed. Data about demographic characteristics, pre-hospitalization baseline conditions, use of antimicrobials, and incidence of both co-infections and secondary infections during hospital stay were collected: these were entered into a computerized database before a statistical analysis was conducted. Calculations and analyses included:

- The patients’ age (with stratification by age-group) and sex.
- The number of days between symptom onset and hospital admission, followed by the length of hospital stay and the days spent in ICU for those patients who required intensive care measures.
- The Charlson Comorbidity Index (CCI) was calculated for each patient. The CCI is a score in which the sum of different comorbid conditions predicts the 10-year survival for a patient. It includes different items, such as myocardial infarction, congestive heart failure, peripheral vascular disease, previous stroke, dementia, chronic obstructive pulmonary disease (COPD), peptic ulcer disease, mild and moderate-to-severe liver disease, diabetes mellitus, hemiplegia, moderate-to-severe chronic kidney disease, localized or metastatic solid tumor, leukemia, lymphoma, and acquired immunodeficiency syndrome (AIDS).
- The administration of active therapies against COVID-19 (namely steroids and remdesivir) was considered for both cohorts, although a small proportions of patients from the first population was exposed to either agent.
- Empirical antibiotic therapy for CAP (i.e., either third-generation cephalosporins or piperacillin/tazobactam, representing the first- and second-line therapeutic options, respectively) was included in the analysis. The administration of azithromycin was instead considered only for the first cohort of patients. Description of antibiotic options for MDR-associated infections (vancomycin, teicoplanin, linezolid, gentamycin, meropenem, ceftazidime/avibactam and ceftolozane/tazobactam) was also described and reported in the analysis.
- The incidence of co-infections, defined as bacterial infection diagnosed at the time of hospital admission (CAP) and secondary infections, defined as bacterial infection diagnosed during hospital stay (namely HAP, VAP, BSI, and/or UTI) and the isolated microorganism, as well as the consequent development of sepsis and septic shock.
- The outcome: discharged or deceased.

### 2.5. Data Analysis

Evaluation and highlight on how the analyzed aspects changed during the two epidemic outbreaks were studied with different statistical tests. Quantitative variables were expressed as median (interquartile range), while categorical variables were expressed as absolute frequencies and percentages. Data were organized into tables and then compared with the Z-proportion test and Pearson’s χ^2^ test for dichotomous and non-dichotomous variables, respectively. Regressive data were instead analyzed with the Wilcoxon regression test. Statistical significance was assumed when *p*-values < 0.005.

### 2.6. Study Definitions

- The date of disease onset was the day when symptoms were first noticed.
- The Third International Consensus Definitions for Sepsis and Septic Shock (Sepsis-3) was used as a reference of sepsis definition [29].
- Secondary infections were considered if clinical suspicion was confirmed with both laboratory alterations of inflammatory indices and microbiological isolation from blood, urinary, and/or respiratory specimens; coinfections are defined as bacterial infections diagnosed at hospital admission; secondary infections are defined as bacterial infection diagnosed during hospital stay.
- Pathogens were considered multi-drug resistant according to the European Committee on Antimicrobial Susceptibility Testing (EUCAST) criteria for antimicrobial susceptibility [28].

## 3. Results

Table 1 represents baseline patients’ characteristics, therapeutic options for COVID-19, and data about the course of hospitalization. The median Charlson Comorbidity Index Score differed extensively between the two groups: the first cluster of patients was characterized by a median CCI of 1 (IQR 0–2), in contrast with a median CCI of 4 (IQR 2–6) in the second one. Active COVID-19 therapies such as remdesivir, intravenous steroids (i.e., dexamethasone), and prophylactic low-molecular weight heparin were introduced as standard-of-care for hospitalized patients who met the specific eligibility criteria for these drugs [26,27]. In-hospital mortality was similar for the two populations (23.5% vs. 24.7%).

In the first phase of the pandemic, in the absence of solid evidence and lack of a scientific consensus, many national and international guidelines recommended that empirical antibiotic therapy for suspected bacterial CAP be started in most patients with COVID-19, according to data describing a high incidence of secondary infections associated with viral respiratory illnesses [30,31,32]. Empirical antibiotic therapy differed profoundly between the two populations. In the first cohort, third-generation cephalosporins were given as empiric antimicrobial therapy to most patients admitted to the hospital with confirmed SARS-CoV-2 infection (70.2%). Other commonly prescribed antibiotics were azithromycin (18%) and piperacillin/tazobactam (34.7%): the latter was allotted for those patients who presented signs of sepsis or septic shock on hospital admission. Contrariwise, the use of third-generation cephalosporins was halved in the second group as data from the first phase of the pandemic suggested a low incidence of COVID-19-associated secondary infections [33,34]. Azithromycin administration was withheld, and the rationale for the use of piperacillin/tazobactam (33.2%) was maintained. While antibiotic therapy was sustained for most patients from the first outbreak, empiric antibiotic therapy was instead either discontinued when secondary infection was ruled out or optimized according to antimicrobial susceptibility of isolated bacteria. 

As shown in Table 2, the rate of secondary infections differed extensively: a total of 8.2% vs. 23.5% was reported, respectively. On hospital admission, first-wave patients tended to present a higher incidence of co-infection with bacterial CAP when compared to second-wave patients, as well as a higher incidence of HAP. The rate of ventilator-associated pneumonia was similar. Bloodstream infections and urinary tract infections were more common in the second group of patients. A higher incidence of sepsis and septic shock was observed in the second cohort, but a statistically significant difference was lacking. Collectively, among MDR pathogens, Gram-negative bacteria were most common. Different incidences of the different pathogens may be observed comparing the two different epidemic waves: a higher incidence of carbapenem-resistant *K. pneumoniae* was reported from first outbreak patients: all isolates were sampled from ICU patients which had been transferred to our institution from the same ward from a different hospital. These cases belong to a larger cluster of KPCs that contributed to the spread of the same pathogen during the first wave of the pandemic in the metropolitan area of Milan, Lombardy region. MRSA was, instead, the most common isolate for second outbreak patients.

As shown in Table 3 and Table 4, a different antibiotic approach was adopted between the two epidemic waves as far as MDROs management is concerned. During the first period of the pandemic, most of the MDR microorganisms were identified as colonizers due to more active surveillance and screening of MDROs: as such, the isolated pathogens were addressed accordingly with prompt infection control measures and were, therefore, less likely responsible for secondary infections. Conversely, for the second population the majority of MDROs was responsible for active infections. MDR antibiotic treatment was comparable for the two populations: all treated isolated of carbapenem-resistant *K. pneumoniae* and DTR-*Pseudomonas* received combination therapy, with ceftazidime/avibactam and ceftolozane/tazobactam in combination with either aztreonam, fosfomycin, or an aminoglycoside as second agent. The only ESBL+ isolated strain from the first population was treated with gentamycin in the setting of UTI; conversely, all ESBL+ isolates from the second outbreak were treated with carbapenems in the setting of BSI. It is important to point out that among first-outbreak patients, only one patient died of MDR-associated infection. Conversely, a much higher incidence is observed for second outbreak patients, where secondary infections impacted extensively on mortality: in line with other studies, this finding may explain the reason why the mortality rates of the two compared populations are superimposable despite the introduction of active therapies against COVID-19 [21,35,36].

## 4. Discussion

The study demonstrated that from a demographic point of view the second population of hospitalized inpatients was older and presented a higher CCI compared to the first. Hospitalization and chronic degenerative comorbidities are recognized risk factors for bacterial colonization (especially by MDROs in a region with a prevalence of such microorganisms) [19]. The latter also contributes to patient fragility, increasing the likelihood of developing invasive infection. These patients are at risk of severe illness requiring the use of catheters (both intravascular and urinary) and may require both invasive and non-invasive ventilation, which are risk factors for the development of secondary infections [37]. Other factors that may contribute to a higher risk of secondary infections are the use of steroids [38,39], which were included in the standard-of-care for the second population, given their protective effect towards COVID-19-associated mortality. Altogether, these characteristics make patients from the second cohort an ideal population for the development of secondary infections (8 vs. 24%).

Bacterial isolates from the two populations are typically nosocomial, especially for second outbreak patients. Factors that may further explain a higher incidence of these pathogens include inadequate adherence of the medical staff to standard hygiene measures and personal protective equipment (PPE) during the pandemic. In emergency settings, ward overcrowding may predominate, with consequent less effective isolation measures among patients. This was particularly true during the second outbreak of the pandemic, when an overall tiredness of the medical staff impacted hugely the quality of both focus and attention on patient care. All described factors add up to an increased rate of pathogenic bacteria cross-transmission among patients, further explaining the higher incidence of these events for the second population [16,18].

Among the different bacterial species that were isolated from the two populations, a higher number of MDRO-associated infections was observed in the second population. In this study, most isolated microorganisms are Gram-negative species, which is line with other studies reporting Gram-negative bacteria to be the most common in COVID-19 patients [10,40]. While second outbreak patients had a lower incidence of carbapenem-resistant *K. pneumoniae* when compared to first outbreak patients, a higher prevalence of ESBL+ *E. coli*, DTR-*Pseudomonas*, MRSA, and VRE was observed. For these patients, most of the reported isolates were treated in the setting of active infection: in contrast, most isolates from first outbreak patients were identified as colonizers. Antibiotic treatment was similar for most MDR isolates in both populations. *K. pneumoniae* was the most frequent isolate in both populations. This is in line with other reports suggesting *K. pneumoniae* to be the most common isolate in COVID-19 patients [18,41]. Infections were treated with a combination therapy regimen, including ceftazidime/avibactam as a first-line agent. Combination therapy was chosen also for DTR-*Pseudomonas*, while ESBL+ *E. coli*, MRSA, and VRE were commonly treated using a single drug regimen.

MDRO-associated death changed significantly between the two populations. During the first epidemic wave, only one patient died of MDRO-associated infection: conversely, the incidence was much higher for second outbreak patients, with an even distribution among the different isolated microorganisms and most deaths being caused by DTR-*Pseudomonas* infection. Whether this finding reflects patients’ severity or contributes to it is uncertain, but it highlights the complexity of COVID-19 patients [42,43]. The isolation of a bacterium from a COVID-19 patient requires important infection control strategies, as these patients are more at risk of developing invasive infection for several reasons [35]: of note, the immune changes associated with an advanced age, SARS-CoV-2 per se and the use of steroids may favor development of active infection. The first outbreak of the epidemic was characterized by a younger population and the absence of steroids in COVID-19 management protocols and, importantly, by a more careful adherence to infection control procedures. This allowed for prompt identification and correct management of suspected cases of secondary infections, as can be seen by the relatively low incidence of active infection with respect to colonizers in this study. Contrarily, poor adherence to self-protective measures by the medical staff, low quality of in-hospital infection control, and a high workload predominated during the second outbreak enhanced the transmission of bacteria and development of invasive and often fatal infections in previously colonized patients [44].

This study aimed at describing the incidence and epidemiology of bacterial infections and their antibiotic resistance profiles among hospitalized COVID-19 patients, comparing two populations of consecutively hospitalized inpatients belonging to the first and the second outbreak of the pandemic, respectively. Currently, in our institution, screening for MDROs is reserved for patients admitted to the intensive care unit or who have been in close contact with patients with documented MDRO-associated infection. Most patients with an advanced age and chronic degenerative diseases are not suitable candidates for intensive care measures, so the spectrum of MDRO epidemiology in our hospital remains somewhat limited. Therefore, the true in-hospital incidence and, consequently, prevalence of this phenomenon is currently unknown. In Italy, local epidemiology shows a high prevalence of both Gram-positive and Gram-negative MDROs, representing a serious public health concern. Extending the screening for MDROs to other departments could be advantageous, as it could help define transmission dynamics and apply effective infection containment strategies, such as prompt isolation and active surveillance of documented cases, as well as to identify patients with risk factors for invasive infection. Understanding the true epidemiology of MDRO infections in COVID-19 would allow the construction of a hospital-tailored antibiogram that could, in turn, improve patient management and care. In addition, data were collected from two hospitalized populations before the beginning of the vaccination campaign: studies evaluating the incidence of these events in the vaccinated population are required to assess comparisons and identify possible mechanisms associated to the incidence of MDRO-associated infections in COVID-19. Of note, this could help understand whether the higher incidence of these events is to be attributed to the disease itself or, rather, to the emergency events of the pandemic in the different hospitals.

## 5. Conclusions

This study provides a rich dataset about COVID-19-associated secondary infections between the first and the second outbreak of the pandemic, providing a detailed description of a much larger sample than that of most currently available reports. During the first phase of the pandemic, most bacterial strains isolated from patients were colonizers; conversely, the majority from the second outbreak were responsible for active infection due to several reasons, including patients’ clinical characteristics and the poor adherence to standard hygiene measures and infection control protocols. In countries with a high incidence of MDR pathogenic bacteria, screening and active surveillance of MDROs may be helpful to improve COVID-19 patient care, reinforcing stewardship programs for future epidemic outbreaks.

## Figures and Tables

**Table 1:** Demographic and clinical characteristics of COVID-19 patients admitted to the IRCCS Humanitas Research Hospital. Dichotomous variables were analyzed with the Z-proportion test, while non-dichotomous variables were analyzed with Pearson’s χ^2^ test. Regressive variables were analyzed with the Wilcoxon regression test.

	No. (%) Total (*n* = 510)	No. (%) Total (*n* = 643)	*p*-Value
Age, median (IQR) [min–max], y	67 (56–76) [27–94]	73 (61–81) [26–100]	<0.001
Sex			
Male	343 (67.3)	397 (61.7)	
Female	167 (32.7)	246 (38.3)	
Charlson Comorbidity Index Score, median (IQR)	1 (0-2)	4 (2–6)	<0.001
Therapeutic options			
Remdesivir	2 (0.4)	98 (15.3)	<0.001
Steroids	71 (13.9)	578 (90.2)	<0.001
Low molecular weight heparin	407 (79.8)	608 (94.9)	<0.001
Length from symptom onset to hospital admission, median (IQR), days	6 (3–8)	4 (2–7)	0.724
Length of hospital stay, median (IQR), days	9 (6–14)	12 (8–21)	<0.001
Length of stay in hospital ward, median (IQR), days	8 (6–13)	11 (7–19)	<0.001
Length of stay in ICU, median (IQR), days	9 (6–15)	12 (6–22.2)	0.036
ICU admission	72 (14.1)	60 (9.3)	
Outcome			
Discharged alive	390 (76.5)	484 (75.3)	0.667
Died in hospital	120 (23.5)	159 (24.7)	0.617

Abbreviations. IQR: interquartile range; ICU: intensive care unit.

**Table 2:** Rate of secondary infections and isolated MDROs of COVID-19 patients admitted to IRCCS Humanitas Research Hospital. Dichotomous variables were analyzed with the Z-proportion test.

	No. (%) Total (*n* = 510)	No. (%) Total (*n* = 643)	*p*-Value
Secondary infections	42 (8.2)	151 (23.5)	<0.001
Isolated multi-drug resistant organisms (MDROs)			
Extended spectrum β-lactamase *E. coli*	1 (2.3)	3 (2.0)	
Carbapenem-resistant *K. pneumoniae*	9 (21.4)	7 (4.6)	
Difficult-to-treat resistant *P. aeruginosa*	3 (7.1)	5 (3.3)	
Methicillin-resistant *S. aureus* (MRSA)	3 (7.1)	11 (7.3)	
Vancomycin-resistant *Enterococcus* (VRE)	4 (9.5)	9 (6.0)	
Site of infection			
Community-acquired pneumonia, CAP	6 (14.3)	10 (6.6)	
Hospital-acquired pneumonia, HAP	3 (7.1)	4 (2.6)	
Ventilator-associated pneumonia, VAP	10 (23.8)	32 (21.2)	
Bloodstream infection, BSI	11 (26.2)	47 (31.1)	
Urinary tract infection, UTI	12 (28.6)	58 (38.4)	
Sepsis/septic shock	39 (7.6)	60 (9.3)	0.4678

**Table 3:** Treatment options for MDROs during the first phase of the pandemic at the IRCCS Humanitas Research Hospital.

	No (%) Treated Isolated	Antibiotic Used	No. (%) of Combination Therapy	No. (%) of MDR-Associated Deaths
Extended spectrum β-lactamase *E. coli*	1 (100%)	Gentamycin	0%	1 (100%)
Carbapenem-resistant *K. pneumoniae*	2 (22%)	Ceftazidime/avibactam	2 (100%)	0%
Difficult-to-treat resistant *P. aeruginosa*	2 (67%)	Ceftolozane/tazobactam	2 (100%)	0%
Methicillin-resistant *S. aureus* (MRSA)	2 (50%)	Linezolid; teicoplanin	0%	0%
Vancomycin-resistant *Enterococcus* (VRE)	3 (75%)	Linezolid	0%	0%

**Table 4:** Treatment options for MDROs during the second phase of the pandemic at the IRCCS Humanitas Research Hospital.

	No (%) Treated Isolated	Antibiotic Used	No. (%) of Combination Therapy	No. (%) of MDR-Associated Deaths
Extended spectrum β-lactamase *E. coli*	3 (100%)	Meropenem	0%	2 (67%)
Carbapenem-resistant *K. pneumoniae*	7 (100%)	Ceftazidime/avibactam	7 (100%)	3 (43%)
Difficult-to-treat resistant *P. aeruginosa*	3 (60%)	Ceftolozane/tazobactam	3 (100%)	3 (75%)
Methicillin-resistant *S. aureus* (MRSA)	11 (100%)	Linezolid	0%	3 (27%)
Vancomycin-resistant *Enterococcus* (VRE)	6 (67%)	Linezolid	0%	2 (22%)

## Data Availability

All data can be found in the manuscript.
